# Combining Genetic Tools to Determine Lipoprotein(a) Concentrations and Their Associated Cardiovascular Risks Across Different Ethnicities

**DOI:** 10.1016/j.jacbts.2025.101315

**Published:** 2025-07-30

**Authors:** Raul D. Santos

**Affiliations:** Academic Research Organization, Hospital Israelita Albert Einstein, Sao Paulo, Brazil; Lipid Clinic Heart Institute (InCor), University of Sao Paulo Medical School Hospital, Sao Paulo, Brazil

**Keywords:** aortic valve disease, atherosclerosis, ethnicity, genomics, lipoprotein(a)

**Figure:**
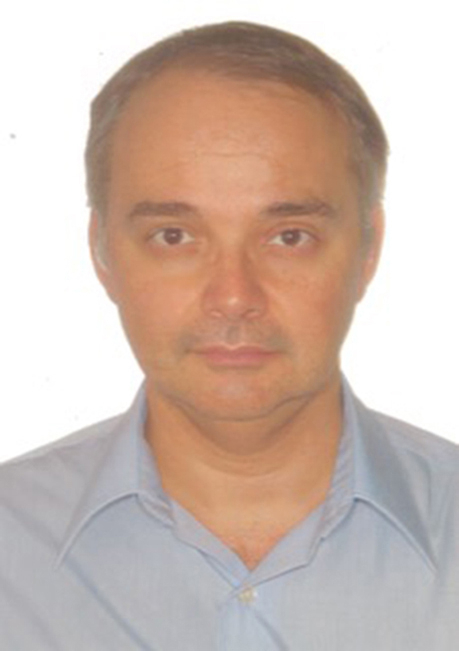

Lipoprotein(a) [Lp(a)] is a cholesterol-rich lipoprotein composed of an low-density lipoprotein particle and an additional apolipoprotein known as apo(a). Extensive epidemiological and genetic studies show an independent association between elevated blood Lp(a) concentrations and both atherosclerotic cardiovascular disease (ASCVD) and aortic valve calcification.^1^^,^^2^ In addition to being cholesterol-rich, Lp(a) is proinflammatory, because it carries oxidized phospholipids. Furthermore, it may also facilitate arterial thrombosis caused by the structural similarity between apo(a) and plasminogen. Lp(a) should be measured at least once in a lifetime. However, most people in the world are unaware of their values.^3^

Blood concentrations of Lp(a) are predominantly genetically determined (90%), and in most individuals, they correlate with the size of circulating apo(a) isoforms.^3^^,^^4^ Larger isoforms tend to become trapped in the liver, where Lp(a) is synthesized, and are therefore associated with lower values. Smaller particles, on the other hand, are linked with higher Lp(a) levels. The major issue with Lp(a) is that approximately 1.4 billion people worldwide have high Lp(a) concentrations, and its population attributable risk to ASCVD is comparable to that of type 2 diabetes.^3^^,^^5^

Unfortunately, high Lp(a) levels can be inherited within families, making them a common genetic trait. However, the inheritance of Lp(a) blood concentrations is a complex process. It varies primarily based on the number of Kringle IV-2 (KIV-2) repeats, a structural component associated with apo(a) size, a few frequent splice site variants (4925G>A or 4733G>A), or single-nucleotide variations (SNVs) in the *LPA* gene.^3^^,^^6^ These genetic traits differ by ethnicity and seem to interact with one another.

In previous epidemiological studies, primarily involving White European populations, KIV-2 repeats have served as proxies for Lp(a) blood concentrations with considerable accuracy.^2^ The SNVs grouped within an *LPA* genetic risk score were also effective predictors of Lp(a) concentrations in Europeans.^6^ However, their value in non-Europeans is questionable caused by heterogeneity in SNV distribution across different populations.^7^

Given the variability in genetic determinants of Lp(a) blood concentrations among individuals of various ethnic backgrounds, it is essential to advance the field and develop tools to assess these factors in diverse populations. In the current study, published in *JACC: Basic to Translational Science*, Telis et al^8^ integrated data from KIV-2 repeats and a previously validated *LPA* genetic risk score^6^ from exome sequencing to estimate Lp(a) concentrations. Importantly, the association with ASCVD and aortic valve events in a diverse U.S. population was also tested. For this purpose, the authors analyzed genetic samples from 76,147 individuals participating in the Helix Research Network. Participants were divided into 2 groups, European and non-European, based on their genomic ancestry. Then, the association between the number of KIV-2 repeats and the Lp(a) genetic risk score was examined in relation to Lp(a) concentrations, the presence of ASCVD, and aortic valve events.

Lp(a) values were available for only 2% of the study population (n = 1,584, of whom 85% were of European ancestry). The polygenic risk score performed well in Europeans, accounting for 40% of the variability in Lp(a) concentration. However, it was not a good predictor for non-Europeans, explaining only 4% of the Lp(a) variability and with poor discrimination.

On the other hand, the KIV-2 copies accounted for 32% and 29% of Lp(a) variability in Europeans and non-Europeans, respectively. Notably, their combination significantly enhanced the prediction of Lp(a) values for both groups of individuals, yielding r^2^ values of 0.49 and 0.34 for Europeans and non-Europeans, respectively. The use of combined genetic tools performed well in identifying individuals with high (>120 nmol/L) and low Lp(a) concentrations across both groups. However, the main limitation of these analyses was that they were conducted on a small population sample, which was even less representative of non-Europeans. This limitation does not solely pertain to the Helix Research Network population; Lp(a) is indeed seldom measured in clinical practice.^3^

Tellis et al^8^ then tested the association of the combined genetic tools with the age of onset of isolated and combined ASCVD events (coronary artery disease, myocardial infarction, peripheral artery disease, ischemic stroke) and aortic valve disease in the entire population, and according to European (n = 61,406) and non-European (n = 14,171) ancestry. The higher values of the combined genetic tools were associated with a shorter time to onset of events compared with the lower ones. A stronger performance was observed in Europeans compared with non-Europeans, likely because of their predominance in the study cohort. Notably, the tools were less robust in identifying aortic stenosis in non-Europeans.

Another interesting finding was that the genetic tools were able to identify individuals at higher risk of events, even among those considered to have a low ASCVD risk caused by the absence of classical risk factors like hypertension, obesity, and diabetes (3% absolute risk of coronary artery disease and 1.5% in those with high and low combined scores, respectively). This aligns with previous evidence indicating an independent association between Lp(a) and ASCVD events. Of course, considering the multifactorial origin of atherosclerosis, the absolute risk of events will be higher among individuals with elevated Lp(a) and other risk factors compared with those with elevated Lp(a) alone.^3^ Finally, an association was observed between the Lp(a) genetic scores and the early onset of coronary artery disease events.

In addition to the relatively small number of Lp(a) determinations, other limitations of the study include the retrospective analysis of ASCVD and aortic valve events, the relative limited number of non-European individuals, the absence of adjustment for all ASCVD risk factors in the Cox models, and the possibility that not all genetic determinants of Lp(a) were tested. Nonetheless, this is a very elegant study, and the authors should be commended for their efforts.

The key finding of the study was the additive value of the KIV-2 score to the SNV risk score, particularly among non-European individuals. Indeed, the SNV score derived from a predominantly white UK population^6^ has limitations in individuals from other ethnicities, considering the heterogeneity of Lp(a) distribution according to race.^1^

Indeed, genetic tools may be an alternative to the conventional determination of blood Lp(a) values and ensuing ASCVD risk, particularly considering technical issues with Lp(a) assays,^1^ especially for epidemiological studies like this one. However, it is essential to note that, currently, there is no evidence that these tools outperform simple Lp(a) testing in predicting ASCVD and aortic valve events.^6^

The study by Tellis et al^8^ adds another piece of information to help solve the many mysteries of Lp(a)^9^; indeed, 2 genetic tools seem better than one in showing an association between Lp(a) and ASVCD events in people of different ethnicities. Hopefully, studies aimed at intensively reducing Lp(a) with RNA-interfering medications will establish its causality in atherosclerosis.^10^ Until results are available, we should measure Lp(a) in everyone, stratify the ASCVD risk using clinical tools, detect subclinical coronary atherosclerosis, and treat any other risk factors present, especially reducing low-density lipoprotein cholesterol intensively in those considered high-risk.^1^

## Funding Support and Author Disclosures

Dr Santos has received honoraria related to consulting, research, and speaker activities from Amgen, Arrowhead, Daiichi Sankyo, Esperion, Ionis, Eli Lilly, Novo Nordisk, Novartis, PTC Therapeutics, Torrent, Sanofi/Regeneron, and Ultragenyx; and is the recipient of a scholarship from Conselho Nacional de Pesquisa e Desenvolvimento Tecnológico, Brazil (CNPq) #303771/2023-2.
